# Effect on β-galactosidase synthesis and burden on growth of osmotic stress in *Escherichia coli*

**DOI:** 10.1186/2193-1801-3-748

**Published:** 2014-12-17

**Authors:** Pushkar Malakar, Vivek K Singh, Richa Karmakar, Kareenhalli V Venkatesh

**Affiliations:** Department of Biosciences & Bioengineering, Indian Institute of Technology Bombay, Powai, Mumbai, 400076 Maharashtra India; Department of Chemical Engineering, IIT Bombay, Mumbai, 400076 India

**Keywords:** *Escherichia coli*, Osmotic stress, Unnecessary Gene Expression, IPTG, Growth rate

## Abstract

Osmotic Shock is known to negatively affect growth rate along with an extended lag phase. The reduction in growth rate can be characterized as burden due to the osmotic stress. Studies have shown that production of unnecessary protein also burdens cellular growth. This has been demonstrated by growing *Escherichia coli* on glycerol in the presence of Isopropyl-β-D-1-thiogalactopyranoside (IPTG) to induce β-galactosidase synthesis which does not offer any benefit towards growth. The trade off between osmotic stress and burden on growth due to unnecessary gene expression has not been enumerated. The influence of osmotic stress on β-galactosidase synthesis and activity is not clearly understood. Here, we study the effect of salt concentration on β-galactosidase activity and burden on growth due to unnecessary gene expression in *E.coli*. We characterize the burden on growth in presence of varying concentrations of salt in the presence of IPTG using three strains, namely wild type, ∆lacI and ∆lacIlacZ mutant strains. We demonstrate that the salt concentrations, sensitively inhibits enzyme synthesis thereby influencing the burden on growth. In a wild type strain, addition of lactose into the medium demonstrated growth benefit at low salt concentration but not at higher concentrations. The extent of burden due to osmotic shock was higher in a lactose M9 medium than in a glycerol M9 medium. A linear relationship was observed between enzyme activity and burden on growth in various media types studied.

**Electronic supplementary material:**

The online version of this article (doi:10.1186/2193-1801-3-748) contains supplementary material, which is available to authorized users.

## Introduction

External stress, such as osmotic shock, is known to burden the growth of the cell, wherein resources are channeled towards adaptation, thereby reducing the growth rate (Csonka 1989; Record et al. 1998). Osmotic stress is known to affect the phenotypic properties of cells such as metabolism, growth and protein synthesis. An increase in the external osmolarity causes loss of water from the cell resulting in shrinkage and arrest in the cell division (Record et al. 1998). The cells synthesize internal osmolytes, such as glycerol or trehalose, to restore cell volume and resumes cell growth post adaptation (Shabala et al. 2009). Thus adaptation is defined based on the restoration of cell division after an initial extended lag in growth on exposure to salt concentration resulting in osmotic stress. While the effect of salt concentration on growth and metabolism is well studied, the effect of salt concentration on enzyme synthesis and activity has not been characterized. There is a study reported wherein the authors demonstrate that viable but non-culturable cells of *Escherichia coli* retain enzyme activity and enteropathogenicity when exposed to sea water (Davies et al. 1995; Pommepuy et al. 1996). To address this issue, we focus our study on the effect of salt concentration on burden to growth and β-galactosidase synthesis or activity in *E.coli.* Further, we characterize the effect of salt concentration on cost phenomenon in a glycerol medium by inducing β-galactosidase synthesis using IPTG. Note that the synthesis of β-galactosidase in a glycerol medium does not offer any growth advantage and is an unnecessary protein in the absence of lactose (Malakar 2014; Malakar & Venkatesh 2013; Malakar & Venkatesh 2012).

It has been shown that the production of unnecessary protein burdens the cell and reduces the growth rate (Novick & Weiner 1957; Horiuchi et al. 1962; Andrews & Hegeman 1976; Koch 1988; Nguyen et al. 1989; Dong et al. 1995; Dekel & Alon 2005; Alon 2006; Malakar & Venkatesh 2014). This reduction in growth rate characterizes the cost to the cell, where the resources are channeled for the synthesis of the unnecessary protein (Maaloe & Ole 1966; Vind et al. 1993; Alon 2006). Studies have indicated that the reduction in growth rate due to unnecessary gene expression or the cost phenomenon depends on the transcriptional efficiency, ribosomal capacity and the quality of medium used for growth (Scott et al. 2010; Malakar & Venkatesh 2012; Malakar 2014). However, if the enzyme synthesized helps in the metabolism of a substrate, the enzyme synthesis provides benefit to the cell. Thus the organism has to balance the cost and benefit due to the synthesis of protein. The trade off between cost and benefit is a fundamental aspect of selection by evolution (Dekel & Alon 2005; Alon 2006). This trade off also determines which regulatory circuit will be selected in a given environmental condition (Dekel et al. 2005; Babu & Aravind 2006; Camas et al. 2006; Zaslaver et al. 2006; Kalisky et al. 2007; TÇŽnase-Nicola & Ten Wolde 2008). Understanding protein cost and adaptation is also important in biotechnology industries where micro-organisms are used to produce gratuitous proteins (Shachrai et al. 2010). Expression of unnecessary genes is one type of stress, which a cell faces within itself. Apart from this cells are exposed to a variety of environmental fluctuations. Cost benefit Analysis is an analysis of energy diversion. The impact on growth of unnecessary protein synthesis (cost) and necessary protein synthesis (benefit) under osmotic stress condition had not been reported in the literature.

It has been demonstrated that in *Escherichia coli*, the lac operon operates optimally to a given lactose concentration to achieve a balance between cost and benefit (Dekel & Alon 2005). The lac operon in *E.coli*, a well characterized system, is commonly used to characterize the impact of unnecessary gene expression on growth. *E.coli* is grown on a medium containing glycerol with IPTG, a non-metabolizable inducer of lac operon, synthesizing β-galactosidase which offers the burden on growth without any benefit.

In the current study, we address the effect of salt concentration on β-galactosidase synthesis and activity in *Escherichia coli* and the burden on growth. Characterization of burden on growth due to unnecessary protein production under osmotic shock will provide insights into the effect of protein synthesis and salt concentration on burden. We further analyze the affect of salt concentration on the growth of *E.coli* on lactose, thereby characterizing the benefit experienced by the cells. The study demonstrated that protein synthesis and activity is severely affected by osmotic stress thereby influencing the burden. Comparison of growth in media with and without salt yielded individual contributions of protein synthesis and salt on burden to growth. The stability of synthesized GFP at various salt concentrations, clearly demonstrated that osmotic stress repressed enzyme synthesis and not the activity. Further, a new perspective on adaptation based on protein synthesis is obtained instead of a definition based on cell division.

## Materials and methods

### Strains, media and reagents

The strain *E.coli* MG1655 (WT) CGSC 6300 was a kind gift from Dr. Manjula Reddy, CCMB, India (Samaluru et al. 2007). DMS269 with genotype lacI^**−**^ and DMS1346 with genotype lacI^**−**^ ∆lacZ was obtained from Daniel Stoebel (Stoebel et al. 2008). BL21(DE3) which is tagged with GFP on the lacZ promoter was also used (Davies et al. 1995). All the experiments were done in M9 defined medium consisting of M9 salts, 1 mM MgSO_**4**_ , 0.1 mM CaCl_**2**_, 0.1% glycerol, 0.200 mM IPTG and specified concentrations of glycerol (Merck). The **Z Buffer** (pH 7.0) contained: 60 mM Na_2_HPO_4_, 40 mM NaH_2_PO_4_, 10 mM KCl, 1 mM MgSO_4_, 50 mM 2-mercaptoethanol. **ONPG** (pH 7.0) contained: 40 mg ONPG dissolved in 10.0 ml of **0.1 M potassium phosphate buffer.** For the cost experiment specified concentrations of IPTG obtained from (Invitrogen) were used. Lactose Monohydrate obtained from Himedia was also used in some experiments.

### Growth rate measurements

The exponential growth rate was measured by growing the strains in 50 ml culture in 250 ml flask. These flasks were incubated on shaker at 37**°** C at 240 rpm. Samples were taken and reading carried out at 595 nm using ELISA Reader (BioRad). Regarding error analysis, the data is a mean of 3 or 4 experiments and the maximum error in the growth data was 5%. Specific growth rate was calculated using the following formula.

$$\frac{dX}{dt}=\mu X$$

Where dX/dt = the growth rate of the biomass mg/L t-1

X = the concentration of biomass, mg/L

*μ* = the maximum specific growth rate constant, t-1

This relationship applies for the log-growth phase, when there are sufficient nutrients for growth and when the bacteria have been acclimated to the system. A minimum of four time points in exponential growth phase was used to calculate the specific growth rate. The slope of the linear fit gave growth rate.

### Beta galactosidase assay

Cells were grown on M9 medium with glycerol as the carbon source. Aliquots of culture were taken at fixed OD. The cells were centrifuged and resuspend in 1 ml Z-buffer and were later placed on ice. The OD of cell suspension was measured at 600 nm. 80 μl of 0.1% SDS and 160 μl of chloroform were added to each tube. The tube was vortexed for 15 seconds. The reaction mixture was incubated at 30**°** C for 15 minutes. 160 μl of 4 mg/ml ONPG was added and vortexed well for 10 sec and further incubated at 30**°** C and timed. The reaction tube was removed after about 10 minutes. The reaction was quenched by adding 400 μl of 1 M sodium carbonate. The cell debris was spinned down. The O.D. of the aliquot was measured at 420 nm. The Miller Units was calculated using the following formula: U = 1000 x [(OD420) / [(Time) x (Vol) x OD595] where Vol is volume of the culture used in the assay in mls, and Time is in minutes (Miller 1972). The data is a mean of 3 or 4 experiments and the maximum error in β galactosidase measurement was 10%.

### Determination of burden on growth (ф)

The burden was evaluated by calculating the relative drop in growth rate relative to the maximum growth rate observed in the absence of salt or IPTG. The burden due to salt concentration (фs) was defined as follows.

$$\varphi_{s}=1-\frac{\mu_{G}}{\mu_{G}{{}_{,}}_{\text{max}}}$$

Where, **μ**_**G**_ is the growth rate in a glycerol medium containing salt and **μ**_**G**_**,**_**max**_ is the growth rate on glycerol medium in the absence of salt. The burden due to both salt and the synthesis of unnecessary protein (фt) was evaluated in a similar manner.

$$\varphi_{t}=1-\frac{{\mu_{G}}_{I}}{\mu_{G}{{}_{,}}_{\text{max}}}$$

Where, **μ**_**GI**_ is the growth rate in a glycerol media containing both salt and saturating amount of IPTG (200 μM).

The burden only due to the synthesis of unnecessary protein (фu) was evaluated by subtracting the burden due to salt from the total burden due to both the factors.

$$\varphi_{u}=\varphi_{t}-\varphi_{s}$$

In order to evaluate the benefit offered by lactose in the medium, cells were grown in medium containing lactose (1 mM) and glycerol (1 g/L) at various salt concentrations. The benefit to growth was determined as follows

$$B=\frac{\mu_{L}-\mu_{G}}{\mu_{G}}$$

Where, **μ**_**L**_ is the growth rate on medium containing lactose and glycerol and **μ**_**G**_ is the growth rate on medium with glycerol in the medium at various salt concentrations.

## Results

**Figure 1:**
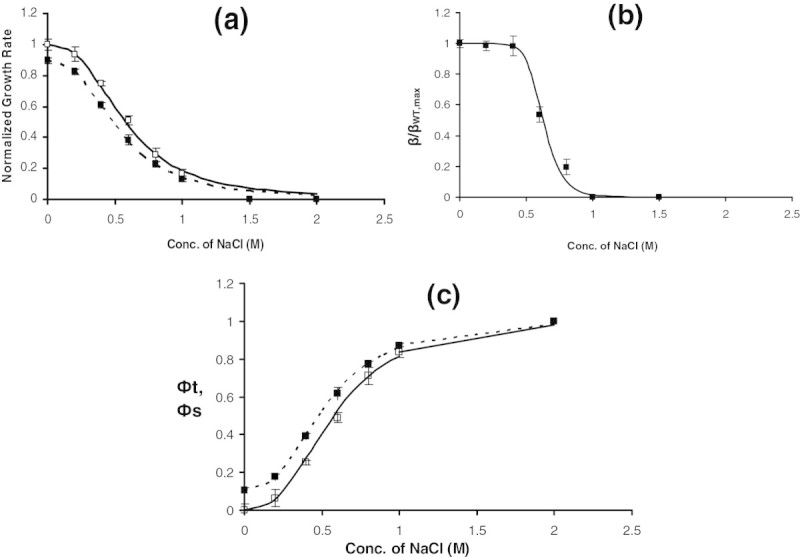
**Effect of NaCl concentration on the growth of** ***E.coli*** **at a fixed glycerol concentration of 1 g/L with and without 200 μM of IPTG: a. Specific growth rate at various NaCl concentrations.** Filled and open Square, respectively, represent the growth rate at various salt concentrations with and without IPTG in the medium. Solid and dashed line represents Hill equation fit for growth rate with and without IPTG in the medium. The specific growth rate was normalized by μ_max_ = h^−1^ for growth of WT on glycerol medium lacking salt. **b**. β-galactosidase enzyme activity at various salt concentrations for growth on medium with IPTG. The β-galactosidase activity was normalized by the maximum observed in the WT strain in a medium lacking salt. **c**. Burden, the relative reduction in growth rate or ф at various NaCl concentrations with and without saturating amount of IPTG in the medium. Filled and open Square, respectively, represent the ф at various salt concentrations with and without IPTG in the medium.

In order to characterize the effect of salt concentration on growth, *E.coli* was grown in a medium containing different salt concentrations and the growth rate was estimated (see Figure 1a). As expected, due to osmotic stress, the growth rate decreased with increasing salt concentration. A Hill equation fit demonstrated that the effect on growth was highly sensitive with a Hill coefficient of 2.7 and a half saturation constant of 0.57 M salt concentration. Experiments were further performed in media containing salt and saturated amounts of IPTG. In this case, in addition to the effect of salt on growth, the effect of unnecessary protein synthesis due to β-galactosidase synthesis also influences growth (see Figure 1a). It can be noted that the growth rate was lower than that observed for growth in a medium with salt alone. A Hill equation fit demonstrated a similar sensitive response with a Hill coefficient of 2.6; however, there was a decrease in both the maximum growth rate from 0.42 h^−1^ to 0.37 h^−1^ and in the half saturation constant from 0.57 M to 0.52 M salt concentration. This indicated that unnecessary protein synthesis was imparting additional burden on growth in osmotic stress condition. Figure 1b shows the β-galactosidase activity observed at various salt concentrations when grown on a medium containing saturating amounts of IPTG. It is clear from the plot that the enzyme activity is strongly inhibited due to the osmotic stress. Normal amounts of β-galactosidase activity was observed upto 0.4 M salt concentration and decreased steeply beyond 0.4 M salt concentration. A Hill equation fit characterizing the response of enzyme activity indicated a highly ultrasensitive response with a Hill coefficient of 9 and a half saturation constant of 0.63 M salt concentration.

**Figure 2:**
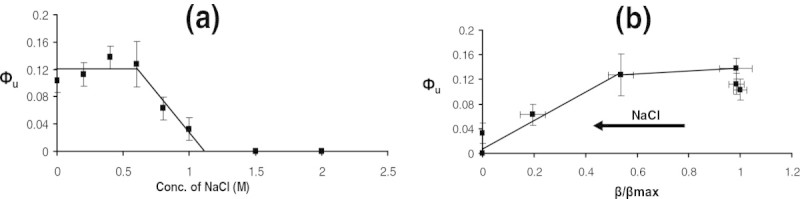
**Burden due to unnecessary gene expression (ф**_**U**_**): a. ф**_**U**_ **at various NaCl concentrations. b**. ф_U_ as a fraction of β-galactosidase activity. Note β-galactosidase activity (β) is normalized with the maximum enzyme synthesized in absence of salt under saturating IPTG concentrations.

The quantification of the growth rate can be used to determine the burden defined as the relative decrease in the growth rate relative to the maximum observed in absence of both the salt and IPTG concentrations (see Materials and Methods section). The **ф**_**s**_ calculated for medium containing various salt concentrations without IPTG showed an increasing trend with increasing salt concentrations before saturating to a value one indicating no growth at high salt concentrations (see Figure 1c). A samilar trend was observed for **ф**_**t**_ the burden noted for growth on a medium with both salt and saturating amount of IPTG, though with a higher value indicating that the burden due to unnecessary protein synthesis was over and above that from salt alone. The value of фs obtained from experiments using media containing salt alone was subtracted from that containing both salt and IPTG (**ф**_**t**_), to determine the contribution of burden due to IPTG alone (**ф**_**u**_). The burden due to the synthesis of unnecessary protein, **ф**_**U**_ , was about 12% in the absence of salt and remained at 12% until 0.6 M salt concentration (see Figure 2a). The contribution of unnecessary protein synthesis to burden decreased beyond 0.6 M salt concentration and was zero beyond 1 M salt concentration (see Figure 2a). This indicated that the cell could adapt perfectly upto 0.6 M salt concentration beyond which the enzyme activity was perturbed due to osmotic stress. This was also confirmed by plotting the burden due to IPTG alone with respect to the normalized β-galactosidase activity. The burden due to enzyme synthesis was about 12% until 50% of the maximum enzyme activity beyond which the burden decreased at higher salt concentrations (see Figure 2b).

**Figure 3:**
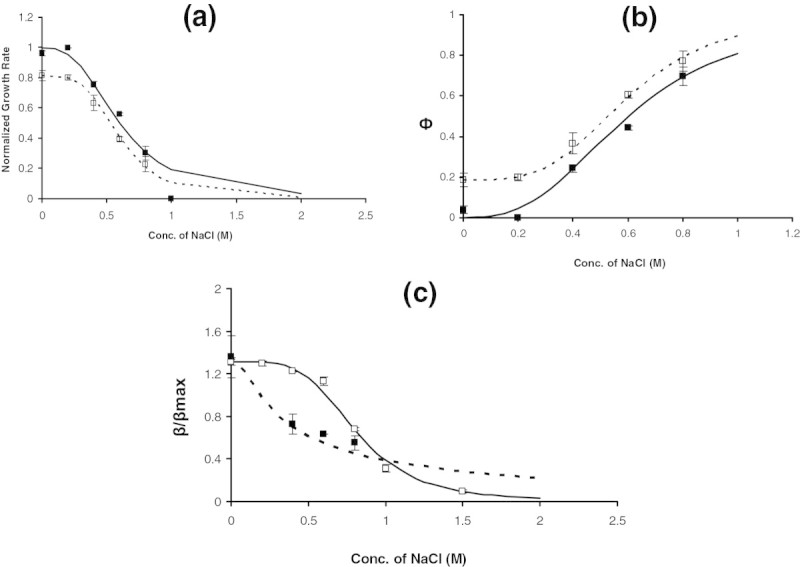
**Effect of NaCl concentration on the growth of ∆lacI and ∆lacIlacZ** ***E.coli*** **at a fixed glycerol concentration of 1 g/L. a.** Specific growth rate at various NaCl concentrations. Filled and Open Square, respectively, represents the growth rate for ∆lacIlacZ and ∆lacI at various salt concentrations. Solid and dashed line represents Hill equation fit for growth rate. **b**. Burden or ф at various salt concentrations. Filled and Open Square respectively, represents the ф for ∆lacIlacZ and ∆lacI. **c**. β-galactosidase synthesis and activity at various salt concentrations. Filled Square represents synthesis and activity while open square represents only activity.

A question that arises from the above analysis is whether the enzyme activity alone is affected by the salt concentration or the enzyme synthesis machinery is also disturbed due to osmotic stress. To address this issue, a mutant *E.coli* cell lacking the lac repressor protein (i.e. ∆lacI strain) was grown on a glycerol medium. This ensures that the β-galactosidase synthesis is constitutive and is independent of IPTG concentrations in ∆lacI strain. As control, a mutant strain lacking both lac repressor and β-galactosidase (∆lacI lacZ) strain was also grown at various salt concentrations. Figure 3a shows the growth rate of the two mutant strains on glycerol at various salt concentrations. The ∆lacI lacZ strain demonstrated a similar behavior as that of a Wild type with a Hill Coefficient of 2.8 and half saturation constant of 0.57 M salt concentration. Note that this was the same value as that for the wild type. This is expected as the ∆lacI lacZ strain would not synthesize

β-galactosidase and only the effect of salt would be observed as in a WT strain. The ∆lacI strain, on the other hand, demonstrated a lower growth rate in the absence of salt. Note that there is no requirement of IPTG (an inducer) in this case as ∆lacI strain constitutively synthesizes β-galactosidase. Although the trend was similar to the WT, the ∆lacI strain demonstrated lower growth rate relative to that observed for WT at all salt concentrations (Figure 3b). A Hill Coefficient of 3.73 and a half saturation constant of 0.6 M salt concentration was noted with a maximum specific growth rate of 0.34 h^−1^. Figure 3c shows the β-galactosidase activity for the constitutive synthesis of the enzyme by ∆lacI strain at various salt concentrations. The constitutive expression of β-galactosidase in ∆lacI synthesized about 40% excess β-galactosidase over the maximum enzyme activity observed in the WT strain. It was noted that the enzyme activity decreased with salt concentration with a Hill Coefficient of 1 and half saturation constant of 0.3 M salt concentration. This indicated that the observed drop in the enzyme activity in ∆lacI was not as steep as that in the WT strain. However, the 50% decrease in enzyme activity was observed at a lower salt concentration in the mutant strain than in the WT strain. To ascertain, if the drop in the activity is due to the synthesis or due to the inactivation of the synthesized protein, β-galactosidase was extracted from ∆lacI cells grown in glycerol in the absence of salt and its activity was measured by exposing it to different salt concentrations for half an hour. Figure 3c shows the deactivation of the enzyme at various salt concentrations with a Hill Coefficient of 4 and half saturation constant of 0.8 M of salt. Thus, the activity of the enzyme remains unaffected until 0.6 M salt concentration and decreases steeply beyond 0.6 M concentration. This clearly demonstrates that the osmotic stress affects both the synthesis of β-galactosidase and its activity.

**Figure 4:**
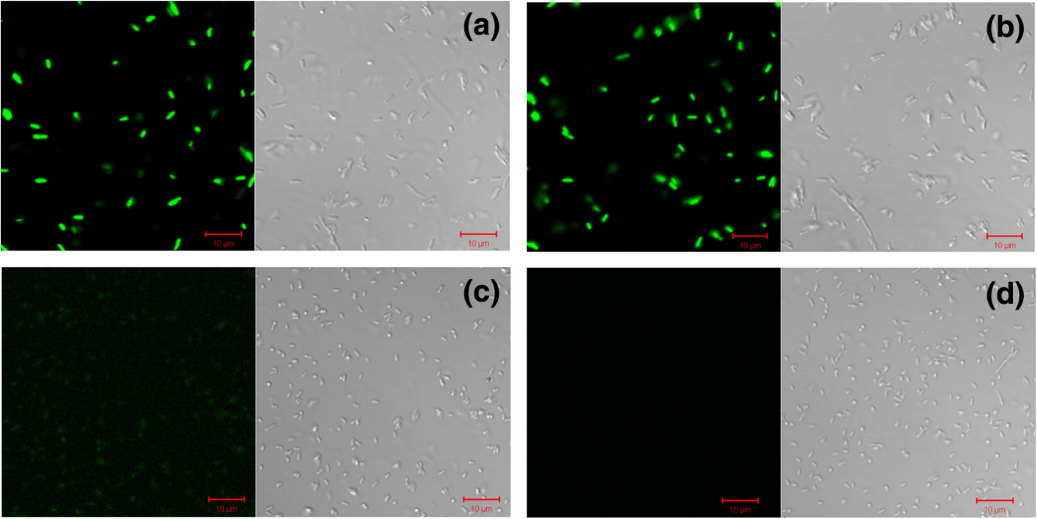
**Fluorescence images capturing the effect of the hyper-osmotic stress on the expression of GFP through the** ***lacZ*** **operon. (a)** GFP expression in cells grown on glycerol in the absence of salt. **(b)** GFP expression in cells grown on glycerol in the absence of salt and later exposed to 1.5 mol l^−1^ salt concentration. No significant decrease in the intensity was observed compared to Figure 1a reflecting that GFP activity is not degraded. **(c)** GFP expression in cells grown on glycerol with 1.5 mol l^−1^salt concentration. GFP expression seems to decrease indicating strong inhibitory effect of salt on lac operon induction. **(d)** GFP expression in cells grown on glycerol in the absence of IPTG and salt (control experiment). GFP is not expressed at all in the absence of inducer (IPTG). Note that Figure 1a, b and c were obtained by providing saturating amount of IPTG (200 μmol l^−1^) in the medium. The corresponding bright field image is shown on right side of each condition showing similar number of cells for each case.

In order to provide direct evidence for the activity inhibition by osmotic stress or repressed enzyme synthesis from high salt concentrations, a GFP intergrated Wild Type *E.coli* strain was used. To characterize the effect of salt concentration on protein synthesis, a GFP intergrated Wild Type *E.coli* strain was grown in a M9 medium containing glycerol and 200 μM of IPTG. Figure 4 shows the fluorescence image of cells expressing GFP. Panel (a) shows the fluorescence image for cells growing in the absence of salt concentration. It can be seen that GFP was expressed in all the cells. Panel (b) shows the fluorescence image for cells exposed to 1.5 M salt concentrations after growing the cells in a M9 glycerol medium containing 200 μM of IPTG in absence of any salt concentration. This ensured that the cells expressed GFP in a normal medium and were later exposed to salt concentration to check the effect of salt on the fluorescence. The fluorescence intensity observed after 3 h was similar to that of the cells unexposed to salt indicating that the GFP fluorescence was not affected by salt concentration. To determine the influence of the hyper-osmotic stress on the expression of GFP through the lacZ operon, cells were grown in M9 media containing glycerol, 200 μM of IPTG and 1.5 M salt concentration. Figure 4c shows the image of cells grown in a glycerol medium containg salt from the start of the experiment. It is clear that the cells express GFP only marginally in this case, indicating a strong inhibitory effect of salt on the expression system. Note that this image (i.e. shown in Figure 4c) was obtained after concentrating the cells grown under high salt concentrations in order to maintain the same number of cells in a frame as in the other images. As a control experiment, cells were grown in the absence of salt and IPTG and the images did not show any GFP expression (see Figure 4d). It can be noted that the corresponding bright field image of cells for the respective experiments shown in the right panel, indicated that the number of cells in the frame were almost identical in all the cases. Similar images analyses were obtained for different salt concentrations and a mean normalized fluorescence value were determined.

**Figure 5:**
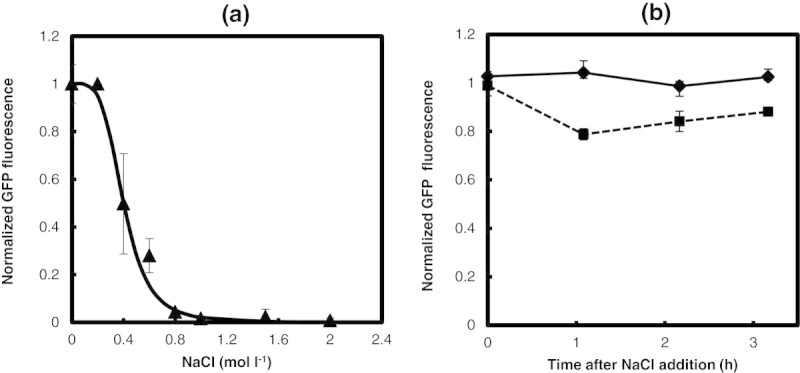
**Effect of NaCl concentration on GFP synthesis and activity. (a)** Normalized GFP fluorescence values measured in WT cells grown in a glycerol M9 medium containing various salt concentrations and saturating amount of IPTG (200 μmol l^−1^). Triangle (▲) and solid line respectively, represents experiment points and Hill equation fit.GFP expression is steeply inhibited with a Hill coefficient of 4.2 and half saturation constant of 0.4 mol l^−1^NaCl. **(b)** Normalized GFP fluorescence values measured in WT cells grown in a glycerol containing M9 medium in absence of and later exposed to 0.2 and 1.5 mol l^−1^ salt concentrations. It can be noted that fluorescence intensity was not affected. Diamond (♦) and square (■) respectively, represents the GFP activity after exposure to 0.2 and 1.5 M NaCl.

The normalized fluorescence value for cells grown in M9 medium with various salt concentrations is shown in Figure 5a. It is clear that GFP expression was strongly repressed by salt concentration. A fit of Hill equation indicated an ultrasensitive repression with a Hill coefficient of 4.2 and a half saturation concentration (K_0.5_) of 0.4 mol l^−1^ salt concentration. To quantify the effect of salt on the GFP activity, experiments were conducted with the cells exposed to 0.2 mol l^−1^ and 1.5 mol l^−1^(see the corresponding Figure 4b) salt concentrations post GFP expression in a normal medium. It was observed that the GFP intensity did not alter even after 3 h on exposure to 0.2 mol l^−1^ salt concentrations, while a decrease of about 15-20% in the intensity was observed on exposure to 1.5 mol l^−1^ salt concentrations. These experiments clearly demonstrated that the expression from the *lacZ* promoter was strongly inhibited by osmotic stress and it was not because of the loss in the fluorescence intensity of GFP. This implied that the protein synthesis machinery was severely affected by the osmotic stress.

**Figure 6:**
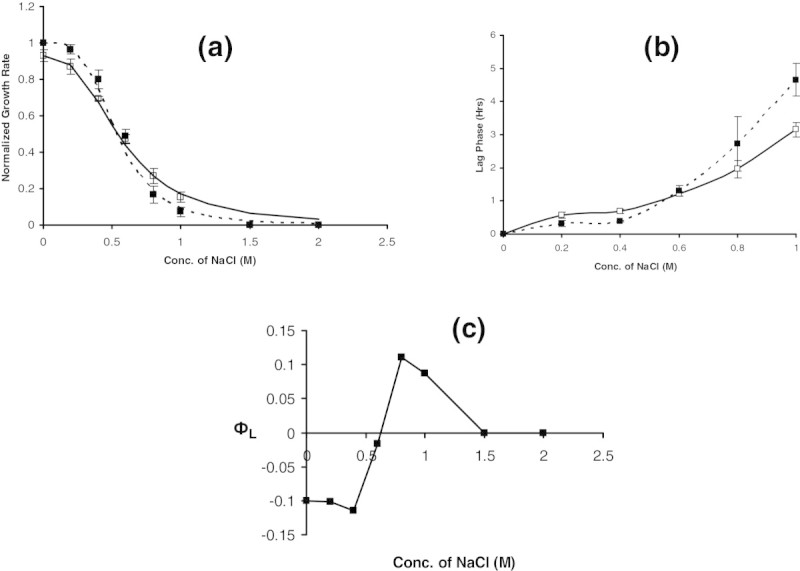
**Growth rate in presence of lactose in the medium: a. specific growth at various concentration of sodium chloride on a glycerol medium (solid line and Open Square) on a glycerol medium with 1 mM lactose (Dashed line and filled square).** Dashed line and solid line represents Hill equation fit. The specific growth rate was normalized by the WT growth rate on lactose (0.45 h^−1^) in a medium having 1 g/L glycerol and 1 mM of lactose in absence of salt. **b**. Lag phase observed for growth on media containing glycerol with and without 1 mM of lactose. Description of data points as in **(a)**. **c**. Burden due to the presence of lactose alone (ф_L_) at various salt concentration. Note that below 0.6 M salt concentration, burden is negative indicating benefit for cells due to the presence of lactose.

The synthesis of β-galactosidase through IPTG while growing on glycerol offers burden characterizing the cost of enzyme synthesis in the presence of salt in the medium. Addition of lactose into the glycerol medium would characterize the benefit offered due to the synthesis of β-galactosidase. Therefore, experiments were performed with a medium containing lactose of 1 mM concentration and 1 g/L glycerol at different salt concentrations. The growth rate at different salt concentrations with and without 1 mM lactose in the medium is shown in Figure 6a. Lactose offered growth benefit upto 0.6 M salt concentration and a higher burden on growth beyond 0.6 M salt concentration. This observation was consistent with the enzyme activity profile in case of growth in a glycerol medium containing saturating amounts of IPTG (Figure 2b) indicating that lower amounts of β-galactosidase was synthesized beyond 0.6 M salt concentration resulting in lower growth without any benefit. This resulted in a sensitive relationship of growth on lactose to salt concentration with a Hill coefficient of 3.6 and half saturation constant of 0.53 M, which was more sensitive than that observed in the absence of lactose (2.7 and 0.57 M, respectively). Figure 6b shows the lag phase observed for the two cases with and without lactose in the medium. The lag phase needed for adaptation also correlated with the growth wherein growth on lactose demonstrated lower lag phase upto 0.6 M salt and higher beyond it. The growth rates determined from the experiments were used to quantify the burden in presence and absence of lactose by evaluating the normalized deviation of growth rate relative to growth in a glycerol medium lacking salt. The contribution to burden from β-galactosidase synthesis due to lactose was evaluating by subtracting the burden due to salt alone from the burden due to salt and lactose. Figure 6c shows the burden due to lactose (ф_L_) as a function of salt concentration. It is clear that upto a salt concentration of 0.6 M, the cells experience benefit. The β-galactosidase activity decreases beyond 0.6 M salt concentration and the benefit reduces leading to a burden. The relationship between the burden due to lactose caused due to β-galactosidase synthesis shows a linear relationship with the β-galactosidase activity.

**Figure 7:**
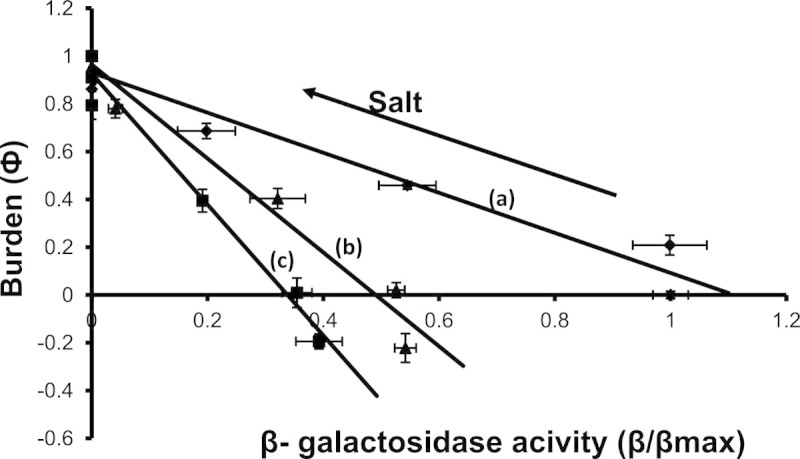
**Burden (ф) as a function of β-galactosidase activity for (a) growth on glycerol + IPTG, filled diamond (b) growth on glycerol + lactose + IPTG, filled triangle (c) growth on glycerol + lactose, filled square.** The lines represent the best linear fit for the three cases. Note that cases **(b)** and **(c)** indicate negative ф representing benefit.

The overall burden experienced by *E.coli* cells in three different media, namely (i) medium containing glycerol and saturating IPTG along with various salt concentration, (ii) medium containing glycerol and lactose with salt and (iii) medium containing glycerol, lactose and IPTG with salt , were compared with the respective normalized β-galactosidase activity (see Figure 7). The burden demonstrated a linear relationship with enzyme activity. It was noted that saturating amounts of IPTG yielded the highest amounts of enzyme concentrations among the 3 media. The media with 1 mM lactose yielded only 40% of the maximum enzyme activity observed in presence of saturating IPTG. Addition of IPTG to lactose yielded higher enzyme activity than lactose alone. This indicated that 1 mM of lactose could not eliminate completely the repression action from lac repressor and therefore the enzyme synthesis was limited. The presence of lactose demonstrated negative burden implying benefit at low salt concentrations. The slopes of the best fit were noted to be negative with values of 0.84, 2.0, and 2.74 for growth on glycerol + IPTG, glycerol + lactose + IPTG and glycerol + lactose, respectively. This indicated that the presence of lactose indicated higher burden to salt concentration as compared to growth in a medium with glycerol alone. Thus, the media and salt concentration had a strong influence on β-galactosidase activity which further determined the extent of burden.

## Discussion

Growth experiments were conducted to determine the burden on growth due to osmotic shock and addition of IPTG leading to β-galactosidase synthesis, an unnecessary enzyme in a glycerol media. Osmotic shock, due to the addition of salt (NaCl), resulted in a reduced growth rate which was highly sensitive indicated by a Hill coefficient close to three. Further, addition of IPTG decreased the growth further due to the burden caused by the synthesis of unnecessary protein. It was observed that enzyme activity was drastically affected due to osmotic shock beyond 0.4 M of salt concentration. Experiments using mutant strains of *E.coli*, namely ∆lacI clearly demonstrated that salt concentrations not only inhibit β-galactosidase activity but also repress its synthesis. The enzyme activity was not affected upto 0.6 M salt concentration. Thus, beyond 0.6 M salt concentration, combination of activity inhibition and repression of synthesis yielded a switch like response of β-galactosidase activity to salt concentration in the WT. The stability of synthesized GFP at various salt concentration clearly demonstrated that the synthesis was repressed and not its activity.

Interestingly, in a ∆lacI strain, although β-galactosidase is constitutively synthesized, the effect of salt was more severe as the repression of synthesis was observed even before 0.6 M salt concentration since the activity is inhibited only beyond 0.6 M salt concentration. This offers a new perspective in the definition of adaptation to osmotic shock. Adaptation is characterized by an extended lag phase before the growth is resumed post adaptation (Parmar et al. 2009). Our experiments using WT and mutant strains of *E.coli* suggests that the enzyme synthesis machinery is strongly affected due to osmotic shock, thus causing a drastic drop in growth rate at higher osmotic shock (beyond 0.6 M salt concentration). Thus, one can infer that the enzyme synthesis may also be a parameter to characterize the extent of adaptation to osmotic shock.

Experiments were also conducted using media containing lactose and various salt concentrations. It was noted that lactose provided benefit only upto 0.6 M of salt, correlating with the normal enzyme activity observed for less than 0.6 M salt concentration. However, beyond 0.6 M salt, due to impaired enzyme synthesis and activity, the growth on lactose reduced. In glycerol environment, expression of beta-galactosidase is unnecessary but in lactose environment expression of beta-galactosidase is necessary for lactose utilization. In the medium with both glycerol and lactose the growth rate was higher than medium with only glycerol at low salt concentrations but at higher salt concentrations the effect is reversed. This effect at higher salt concentration may be due to the effect of beta-galactosidase over expression induced by lactose on the growth rate.

Thus, at higher salt concentration, both salt and the enzyme synthesis caused a cumulative negative effect on growth. The burden at low salt concentration and higher burden at higher salt concentration yielded a highly sensitive response, with a Hill coefficient of 3.6, to growth on lactose in relation to salt concentration. Scott et al. (2010) have reported a phenomenological model relating the effect of transcriptional efficiency, ribosomal capacity and quality of the medium to growth rate (Scott et al. 2010). Their study predicted and experimentally demonstrated a linear relationship between unnecessary enzyme synthesis to extent of burden characterized by lowering of growth rate. Our results confirm the linear relationship between burden on growth due to unnecessary gene expression and enzyme activity under various osmotic stress conditions (see Figure 7). This indicated a limiting ribosomal capacity, when unnecessary protein synthesis is synthesized thereby reducing the proteomic capacity necessary for growth. It appears that the osmotic shock and the unnecessary enzyme synthesis influenced negatively the ribosomal capacity needed for growth.

In summary, the study demonstrated that the burden due to osmotic shock is highly sensitive mainly being affected by limitation in the enzyme synthesis. The effect on enzyme synthesis, which may be limited due to ribosomal capacity, by salt, was switch like. This yielded a new perspective on adaptation, which could be based on enzyme synthesis rather than on the resumption of growth. Further, the benefit by lactose was noted only at low shock levels, namely less than 0.6 M salt concentration. The linear relationship between burden and enzyme activity demonstrated that the net proteomic fraction needed for growth was reduced due to higher osmotic shock. Thus, an analysis of burden on growth in presence of osmotic shock can provide phenomenological insights into the relationship between enzyme synthesis and growth.

## Authors’ original submitted files for images

Below are the links to the authors’ original submitted files for images. Authors’ original file for figure 1 Authors’ original file for figure 2 Authors’ original file for figure 3 Authors’ original file for figure 4 Authors’ original file for figure 5 Authors’ original file for figure 6 Authors’ original file for figure 7
