# Supplementary figures and images for: Effect on β-galactosidase synthesis and burden on growth of osmotic stress in Escherichia coli
Source: Springerplus. 2014 Dec 17;3:748. doi: 10.1186/2193-1801-3-748 (PMC4320194; doi:10.1186/2193-1801-3-748)

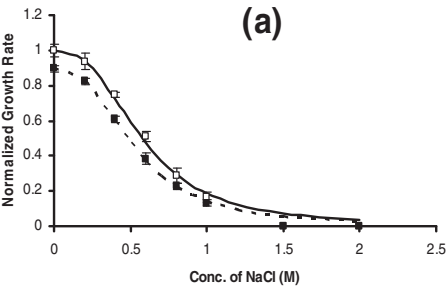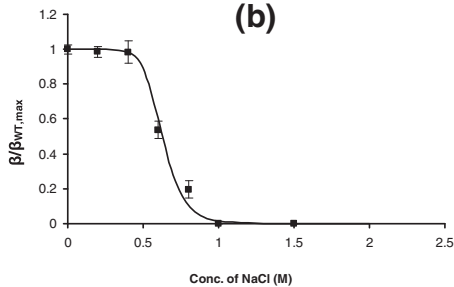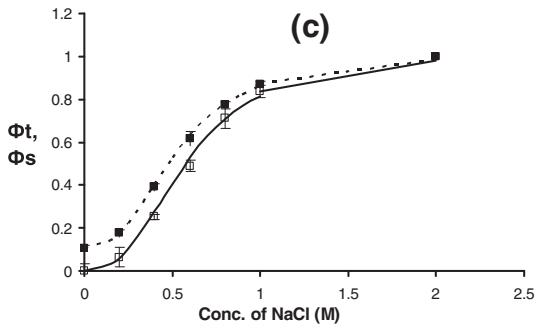

Supplement: Supplementary file 1 — Authors’ original file for figure 1 [file 40064_2014_1521_MOESM1_ESM.pdf]

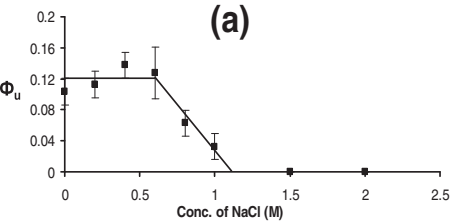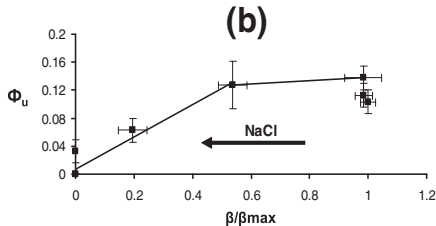

Supplement: Supplementary file 2 — Authors’ original file for figure 2 [file 40064_2014_1521_MOESM2_ESM.pdf]

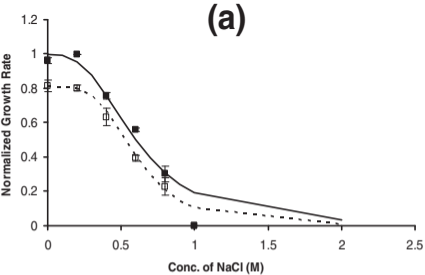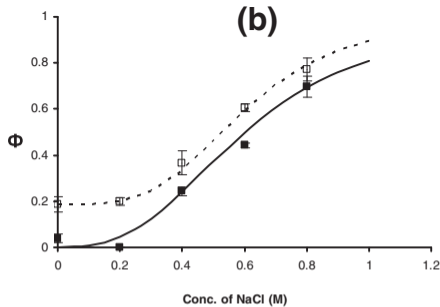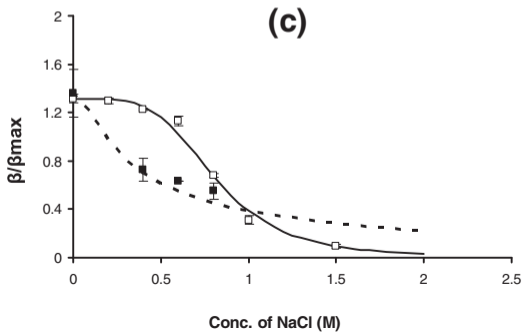

Supplement: Supplementary file 3 — Authors’ original file for figure 3 [file 40064_2014_1521_MOESM3_ESM.pdf]

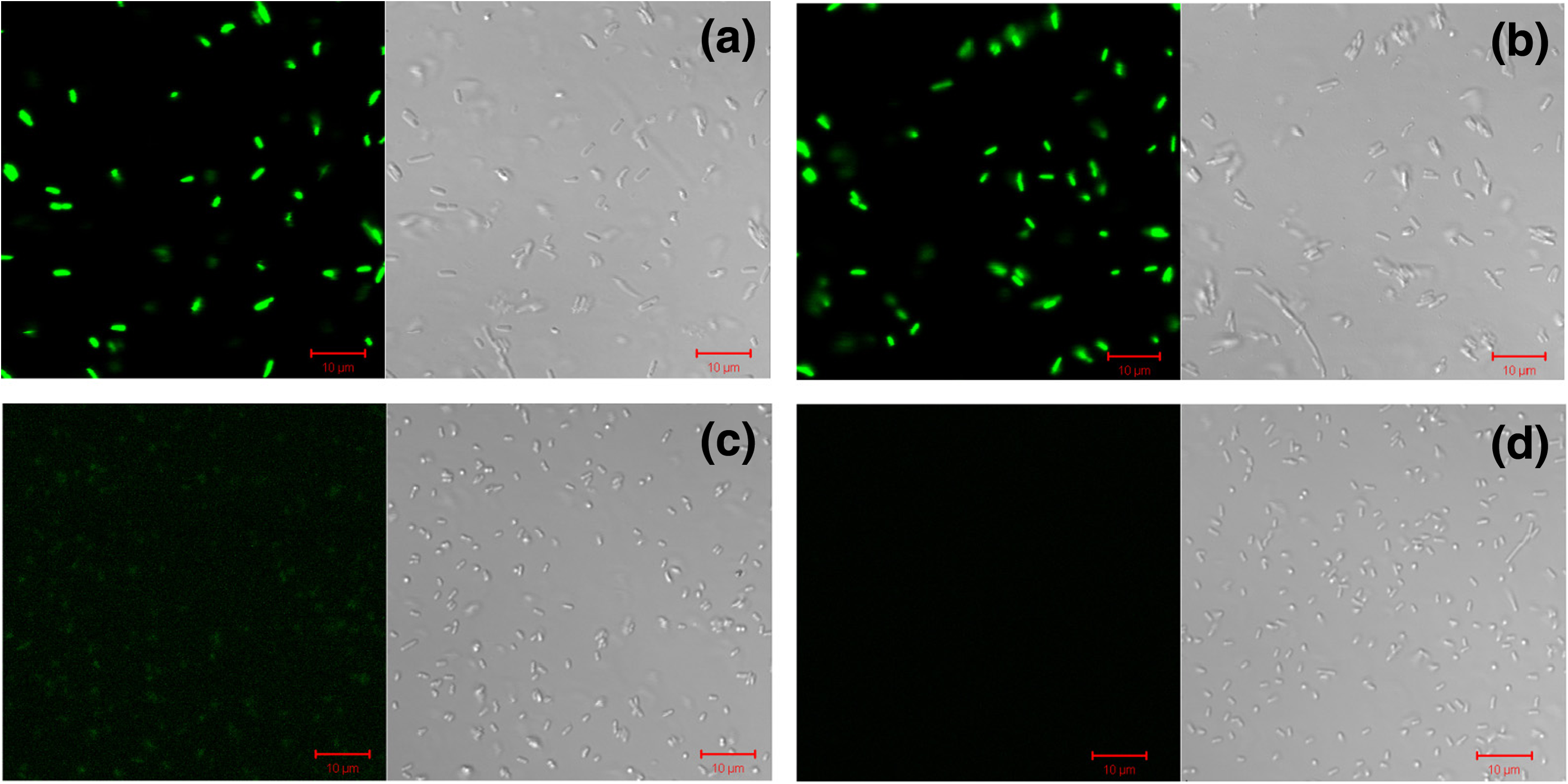

Supplement: Supplementary file 4 — Authors’ original file for figure 4 [file 40064_2014_1521_MOESM4_ESM.tiff]

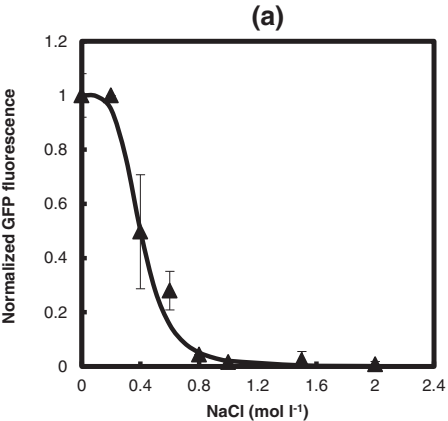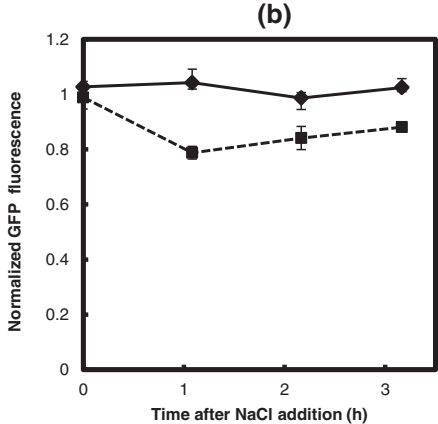

Supplement: Supplementary file 5 — Authors’ original file for figure 5 [file 40064_2014_1521_MOESM5_ESM.pdf]

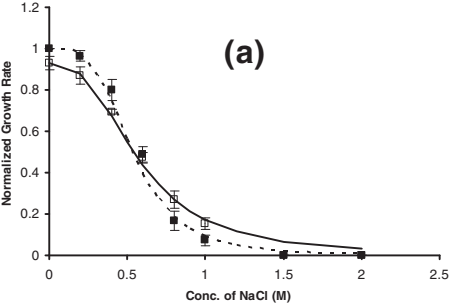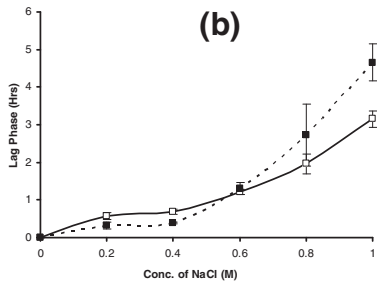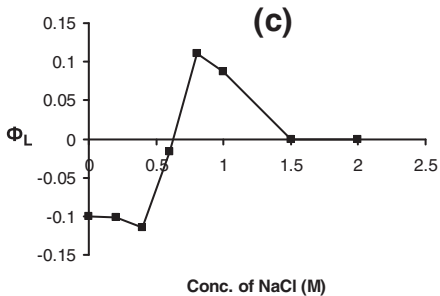

Supplement: Supplementary file 6 — Authors’ original file for figure 6 [file 40064_2014_1521_MOESM6_ESM.pdf]

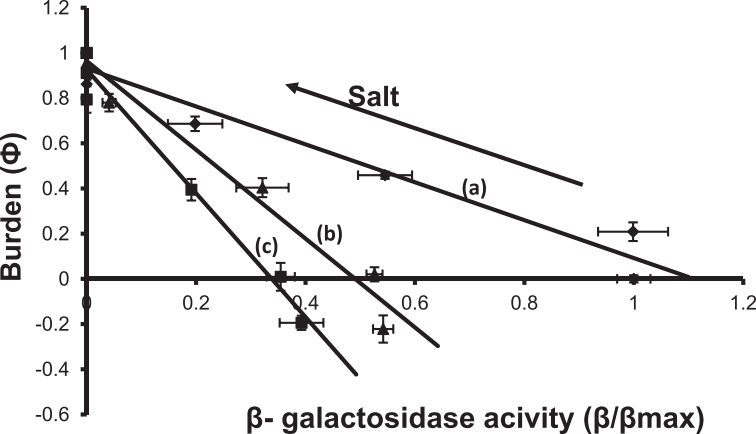

Supplement: Supplementary file 7 — Authors’ original file for figure 7 [file 40064_2014_1521_MOESM7_ESM.pdf]
